# Interaction Between Prediabetes and the ABO Blood Types in Predicting Postsurgical Esophageal Squamous Cell Carcinoma-Specific Mortality: The FIESTA Study

**DOI:** 10.3389/fonc.2018.00461

**Published:** 2018-10-23

**Authors:** Guohui Fan, Dan Hu, Xinran Zhang, Feng Peng, Xiandong Lin, Gang Chen, Binying Liang, Hejun Zhang, Yan Xia, Xiongwei Zheng, Jianzheng Jie, Wenquan Niu

**Affiliations:** ^1^Institute of Clinical Medical Sciences, China-Japan Friendship Hospital, Beijing, China; ^2^Department of Pathology, Fujian Cancer Hospital, Fujian Medical University Cancer Hospital, Fuzhou, China; ^3^Department of Cardiology, The First Affiliated Hospital of Fujian Medical University, Fuzhou, China; ^4^Department of Medical Record, Fujian Cancer Hospital, Fujian Medical University Cancer Hospital, Fuzhou, China; ^5^Department of General Surgery, China-Japan Friendship Hospital, Beijing, China

**Keywords:** esophageal squamous cell carcinoma, prediabetes, the ABO blood type, prognosis, the FIESTA study

## Abstract

**Background:** We aimed to investigate the interaction between prediabetes and the ABO blood types in predicting esophageal squamous cell carcinoma (ESCC)-specific mortality by analysing data from the FIESTA study on normal/prediabetic patients with ESCC.

**Methods:** Total 1,857 normal/prediabetic patients with ESCC who underwent three-field lymphadenectomy between January 2000 and December 2010 and survived hospitalization were analyzable, with follow-up beginning in 2000 and ending in 2015.

**Results:** At the end of the follow-up, there were 1,161 survivors and 696 non-survivors. The follow-up time ranged from 0.5 to 180 months. The cumulative survival rates in normal patients were obviously better than in prediabetic patients. The cumulative survival rates were significantly higher in normal patients than in prediabetic patients for the blood types O and A (Log-rank test *P* < 0.05), while no significance was detected for the blood types B and AB. Adjusted risk estimates for ESCC-specific mortality for prediabetic patients relative to normal patients were statistically significant in the blood type B^−^ group (hazard ratio [HR]: 1.71; 95% confidence interval [CI]: 1.33–2.20; *P* < 0.001), but not in the blood type B^+^ group (HR: 1.12; 95% CI: 0.77–1.64; *P* = 0.5544).

**Conclusions:** Our findings indicate that prediabetes can predict the significant risk of ESCC-specific mortality in Chinese Han patients with the blood types O and A.

## Introduction

Esophageal cancer ranks sixth in cancer-related deaths worldwide, and it is the fourth most common cancer in China (1, 2). As a major type of esophageal cancer, esophageal squamous cell carcinoma (ESCC) is especially prevalent in China (3), because of the aggressive nature of ESCC and its poor survival outcome, the need to identify effective markers involved in the diagnosis, treatment or prognosis for ESCC is urgent. We previously in the Fujian prospective investigation of cancer (FIESTA) study found that hyperglycemia (including both prediabetes and diabetes mellitus) was a predominant risk factor significantly undermining the life expectancy of ESCC patients (4). Given the systematic damages caused by diabetes mellitus can precipitate the development of ESCC (5) and the antitumor potentials of antidiabetic drugs like metformin (6–8), it is of interest to see whether prediabetes can predict the poor prognosis of ESCC after radical resection. However, a literature search has failed to reveal any supportive evidence on this issue.

Evidence is growing indicating the significant association of the ABO blood groups with the risk of both diabetes and cancer (9–14). For example, a large prospective cohort study by Fagherazzi and colleagues showed that people with the O blood type had a lower risk of developing type 2 diabetes mellitus than the other blood types (13). A case-control study by Kumar and colleagues showed reported 1.69-fold significantly increased risk of ESCC associated with the presence of the B blood type (11). In this context, it would be reasonable to speculate that the prediction of prediabetes for postsurgical ESCC survival might be contingent on different ABO blood types.

To prove this speculation, we revisited the database of the FIESTA study by focusing on normal and prediabetic patients with ESCC at admission, aiming to investigate the interaction between prediabetes and the ABO blood group in the prediction of ESCC-specific mortality in Han Chinese patients.

## Methods

### The FIESTA study

The FIESTA study is an ongoing investigation of preoperative factors for predicting disease-specific mortality of common digestive tract cancer, including sites at esophagus, stomach and colon and rectum (4, 15–24). The study proposal was approved by Ethics Committee of Fujian Provincial Cancer Hospital (the current Fujian Cancer Hospital and Fujian Medical University Cancer Hospital). All patients gave written informed consent.

### Study patients

In total, 1,857 normal and prediabetic patients with ESCC underwent three-field lymphadenectomy between January 2000 and December 2010 and survived hospitalization. All study patients were consecutively recruited from the Department of Thoracic Surgery at Fujian Provincial Cancer Hospital. The latest follow-up was completed in December 2015.

### Inclusion criteria

Patients for the first time received surgery for ESCC, which was confirmed by preoperative biopsies or postoperative pathologic analyses. Patients had not received preoperative and postoperative chemotherapy and/or radiotherapy. Patients had no cancer histories besides the non-melanoma skin cancer. Patients with recurrent ESCC and receiving treatment elsewhere were not included.

### Tissue collection

Paired cancerous and adjacent normal tissues were cut during three-field lymphadenectomy, and they were fixed in 10% neutral-buffered formalin for 20 h within 1 h and paraffin-embedded using standard procedures. Clinicopathologic analysis of each tissue sample was completed at the Department of Pathology, Fujian Provincial Cancer Hospital.

### Follow-up assessment

Follow-up was assessed every 6–12 months after discharge for each survivors at the Out-Patient Department, Fujian Provincial Cancer Hospital. If patients didn't show up at scheduled time, they were contacted via phone calls or postal letters. The span from the date of receiving lymphadenectomy to the date of death or the date of the latest follow-up assessment, whichever came first, was recorded in months as the time to event. In fact, all study patients were followed up for over 5 years, making the prediction of survival at 5-year time point accurate.

### Prediabetes definition

Prediabetes was defined according to the diagnostic criteria proposed by the Chinese Medical Association Diabetes Society in 2017 (25), that is, any participant with fasting blood glucose ≥6.1 mmol/L and <7.0 mmol/L, or 2-hour plasma glucose ≥7.8 and <11.1 mmol/L.

### Patient characteristics

At the time of enrollment, each patient was invited to fill in a self-designed structured questionnaire covering information on social demographic and anthropometric characteristics, including birthday, ESCC onset age, gender, the ABO blood type, cigarette smoking status, alcohol drinking status and family cancer history. Cigarette smoking status was grouped into former or current smoking and never smoking. Alcohol drinking status was grouped into former or current drinking and never drinking. A patient was recorded to have a family history of cancer if one or more cases of immediate relatives were diagnosed to have cancer except for non-melanoma skin cancer within three generations.

In addition, body weight and height were measured to calculate body mass index (BMI). Blood pressure was also measured, and hypertension was defined as systolic blood pressure ≥140 mm Hg or diastolic blood pressure ≥90 mm Hg or intake of antihypertension agents.

Clinicopathologic characteristics were obtained from medical charts and pathological reports, including tumor nodes metastasis (TNM) stage (I, II, III and IV) (26), tumor size (in centimeters), depth of invasion (T1, T2, T3, and T4), regional lymph node metastasis (LNM) (N0, N1, N2, and N3), distant metastasis (M0 andM1), histological differentiation (poor, moderate, well) and embolus (positivity and negativity).

### Statistical analyses

Continuous variables were expressed as median (interquartile range), and categorical variables as number (proportion). Two-group comparisons were done by the Mann-Whitney U test or χ^2^-test, where appropriate. The Kaplan-Meier curves and Log-rank tests were used to display and test differences in cumulative survival rates. Interaction analysis and adjusted risk estimates (hazard ratio [HR] and 95% confidence interval [95% CI]) for ESCC-specific mortality were calculated using multivariate Cox proportional hazard regression models. A prognostic nomogram was plotted based on estimates of adjusted multivariable Cox regression models to predict both 3- and 5-year survival rates. The nomogram was realized using “rms” program package in the open-source R software, version 3.5.0 (available at the website: https://www.r-project.org).

All statistical tests were two-sided and probabilities < 0.05 were considered statistical significance. Statistical analyses were done using the SAS software, version 9.4 (SAS Institute Inc.,) unless otherwise indicated.

## Results

### Follow-up records

In this cohort, 1,857 normal and prediabetic patients with ESCC were followed up until December 31, 2015. All patients were aged from 18 to 82 years old, including 1,421 males and 436 females. At the end of the follow-up, there were 1,161 survivors and 696 non-survivors. The follow-up time of all study patients ranged from 0.5 to 180 months.

### Baseline characteristics

Table 1 shows the baseline characteristics of cohort patients. Deaths per 1,000 person-month were 7.4 in patients with the blood type O (*n* = 729), 8.3 with the blood type A (*n* = 530), 8.5 with the blood type B (*n* = 493) and 8.3 with the blood type AB (*n* = 105), respectively. The prevalence rates of hypertension and dyslipidemia were significantly higher in the blood type A group than the others (*P* < 0.05). There was no statistical difference for the other characteristics, including prediabetes, distant metastasis, tumor-node-metastasis (TNM) stage, histological differentiation, embolus, tumor size and body mass index (BMI) (all *P* > 0.05), across four blood types.

**Table 1:** The baseline characteristics of cohort patients according to ABO blood types.

	**ABO blood types**					
**Characteristics**	**O**	**A**	**B**	**AB**	**Total**	***P*-value**
	**(*N* = 729)**	**(*N* = 530)**	**(*N* = 493)**	**(*N* = 105)**	**(*N* = 1857)**	
Deaths, n (per 1,000 person-month)	259 (7.4)	210 (8.3)	183 (8.5)	44 (8.3)	696 (7.96)	0.6004^†^
Prediabetes, n (%)						0.1377
Normal	644 (88.3)	465 (87.7)	416 (84.4)	95 (90.5)	1,620 (87.2)	
Prediabetes	85 (11.7)	65 (12.3)	77 (15.6)	10 (9.5)	237 (12.8)	
Smoking, n (%)	304 (44.2)	196 (39.3)	188 (39.9)	50 (48.5)	738 (41.9)	0.1377
Males, n (%)	552 (75.7)	412 (77.7)	370 (75.1)	87 (82.9)	1,421 (76.5)	0.3037
Age at surgery (years)	55.0 (50.0, 62.0)	56.0 (50.0, 63.0)	55.0 (49.0, 62.0)	56.0 (50.0, 61.0)	55.0 (50.0, 62.0)	0.1823
Drinking, n (%)	134 (19.5)	112 (22.5)	88 (18.7)	25 (24.3)	359 (20.4)	0.3287
Family cancer history, n (%)	91 (13.3)	65 (13.1)	63 (13.5)	20 (19.6)	239 (13.6)	0.3452
Invasion depth, n (%)						0.0826
T1	90 (12.4)	44 (8.5)	59 (12.1)	7 (6.9)	200 (10.9)	
T2	150 (20.7)	104 (20.0)	80 (16.4)	17 (16.7)	351 (19.1)	
T3	388 (53.5)	303 (58.4)	267 (54.8)	59 (57.8)	1,017 (55.5)	
T4	97 (13.4)	68 (13.1)	81 (16.6)	19 (18.6)	265 (14.5)	
Regional lymph node metastasis, n (%)						0.5573
N0	312 (42.8)	218 (41.1)	215 (43.6)	42 (40.0)	787 (42.4)	
N1	216 (29.6)	160 (30.2)	139 (28.2)	32 (30.5)	547 (29.5)	
N2	139 (19.1)	92 (17.4)	103 (20.9)	22 (21.0)	356 (19.2)	
N3	62 (8.5)	60 (11.3)	36 (7.3)	9 (8.6)	167 (9.0)	
Distant metastasis, n (%)						0.7735
Negative	362 (49.9)	250 (48.2)	229 (47.0)	51 (50.0)	892 (48.7)	
Positive	363 (50.1)	269 (51.8)	258 (53.0)	51 (50.0)	941 (51.3)	
Tumor-node-metastasis stage, n (%)						0.1445
I	87 (12.0)	40 (7.7)	54 (11.1)	6 (5.8)	187 (10.2)	
II	238 (32.8)	183 (35.1)	156 (32.0)	39 (37.5)	616 (33.5)	
III	400 (55.2)	298 (57.2)	278 (57.0)	59 (56.7)	1,035 (56.3)	
Histological differentiation, n (%)						0.8608
Well	109 (15.0)	81 (15.3)	80 (16.2)	20 (19.0)	290 (15.6)	
Moderate	496 (68.0)	354 (66.8)	319 (64.7)	66 (62.9)	1235 (66.5)	
Poor	124 (17.0)	95 (17.9)	94 (19.1)	19 (18.1)	332 (17.9)	
Embolus, n (%)	111 (15.2)	89 (16.8)	70 (14.2)	15 (14.3)	285 (15.3)	0.6934
Tumor size (cm)	4.0 (3.0, 5.5)	4.0 (3.0, 5.9)	4.4 (3.0, 5.6)	4.5 (3.0, 6.0)	4.0 (3.0, 5.5)	0.3879
Hypertension, n (%)	137 (18.8)	139 (26.2)	109 (22.1)	26 (24.8)	411 (22.1)	0.0162
Dyslipidemia, n (%)	351 (48.8)	297 (56.7)	260 (53.4)	55 (52.4)	963 (52.5)	0.0485
Number of regional lymph node metastasis	1.0 (0.0, 3.0)	1.0 (0.0, 3.0)	1.0 (0.0 ,3.0)	1.0 (0.0 ,3.0)	1.0 (0.0, 3.0)	0.7682
Body mass index (kg/m^2^)	21.7 (19.9, 24.0)	21.8 (20.1, 24.1)	21.8 (19.8 ,24.2)	22.0 (20.4 ,24.3)	21.8 (19.9, 24.1)	0.6667

*Data are expressed as median (interquartile range) or percentage. P was calculated by the Mann-Whitney U test or the Chi-squared test where appropriate*.

† *P-value was estimated by log-rank test*.

### Overall analyses

**Figure 1:**
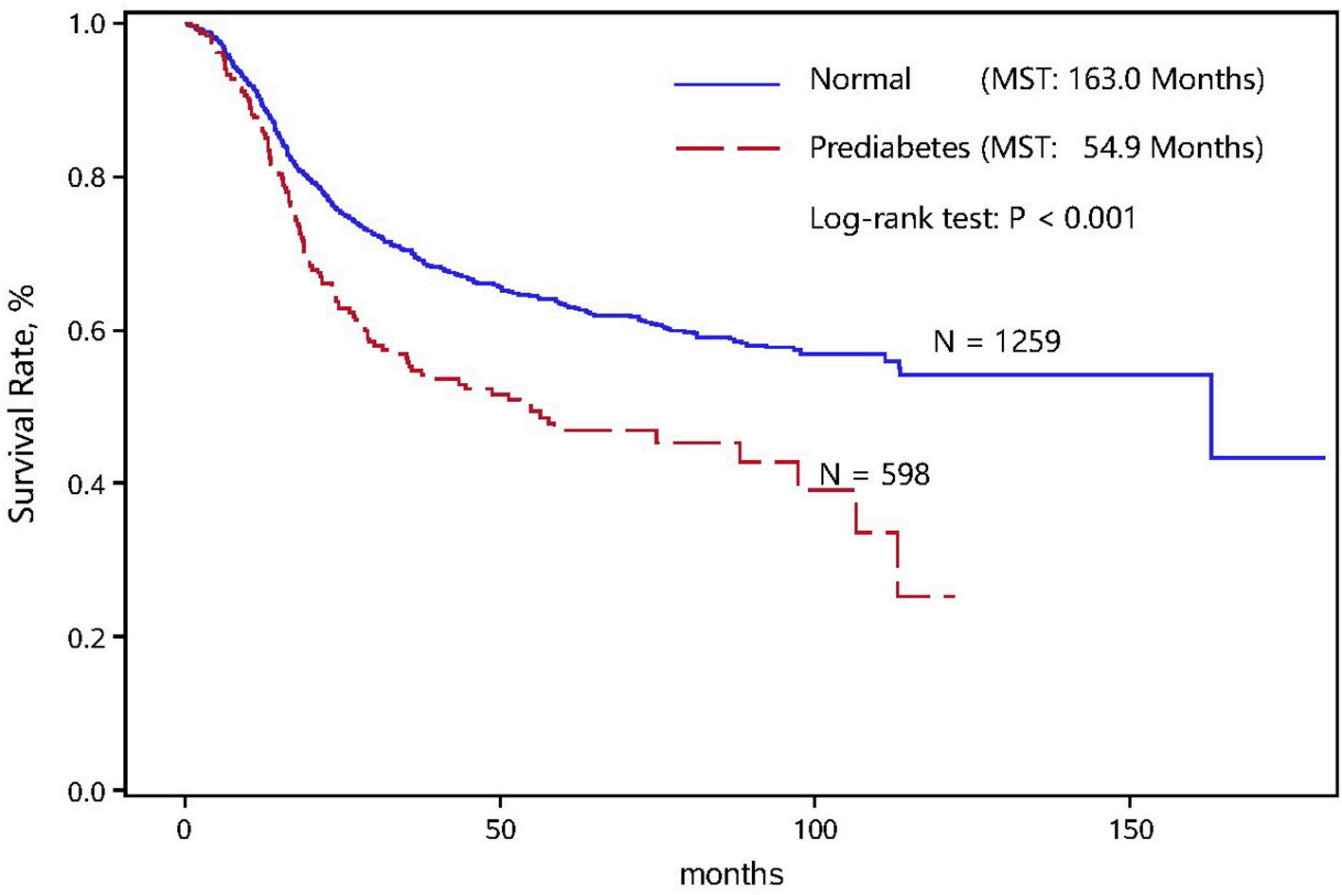
Kaplan-Meier survival curve in normal and prediabetic ESCC patients.

The overall survival status between normal and prediabetic patients with ESCC is shown in Figure 1. The cumulative survival rates in normal patients were obviously better than in prediabetic patients, with significantly longer median survival time (163.0 vs. 54.9 months; Log-rank test *P* < 0.001).

The interaction between prediabetes and the ABO blood group is presented in the Supplemental Table S1. When taking the product of prediabetes and the blood type O as a reference group, the interaction between prediabetes and the blood type B was statistically significant in unadjusted Cox proportional hazard regression model. However, the majority of risk estimates were attenuated after adjusting for gender, smoking, drinking, body mass index, family cancer history, hypertension, dyslipidemia and TNM stage.

**Figure 2:**
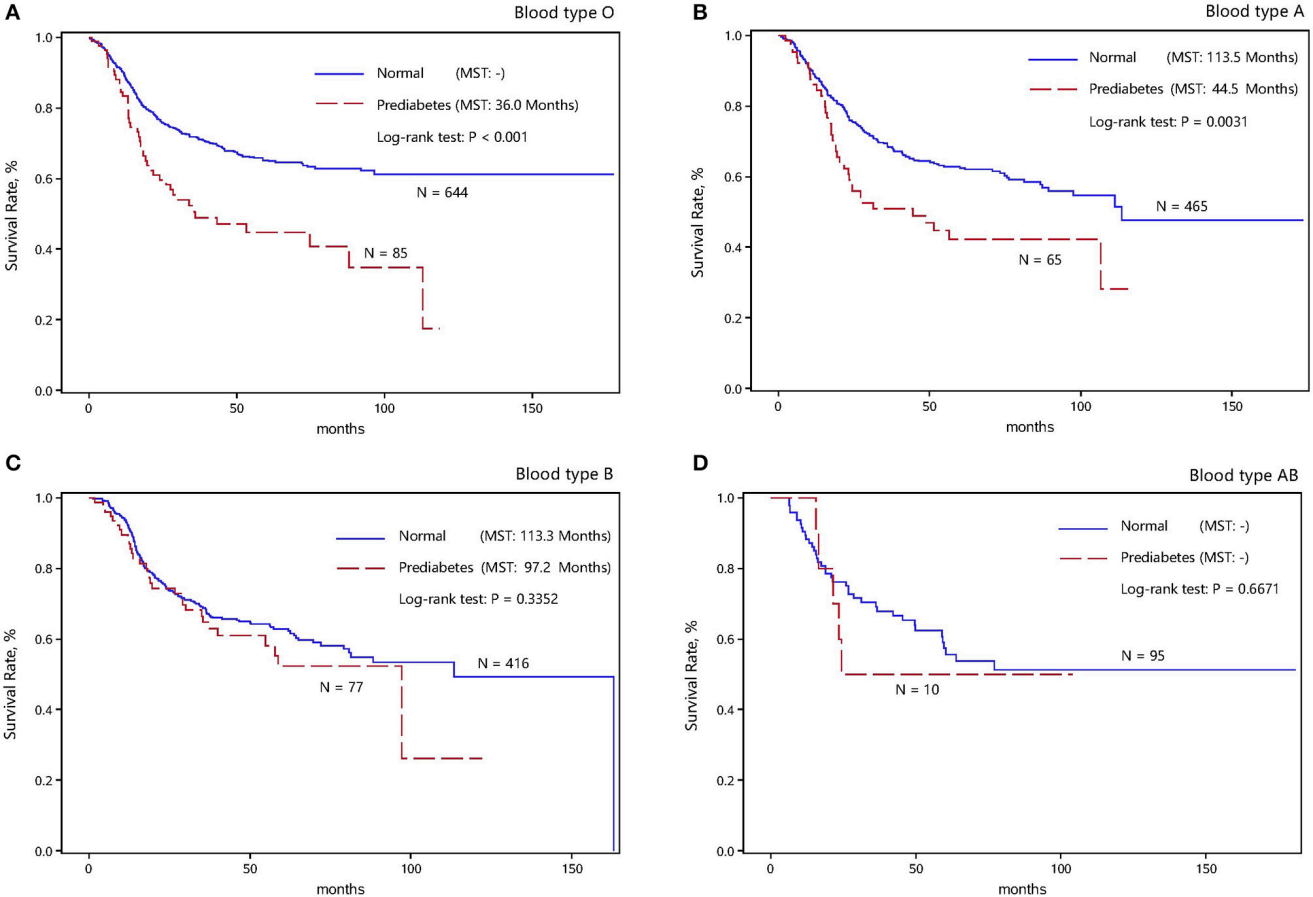
Kaplan-Meier survival curves of in normal and prediabetic ESCC patients with different ABO blood types. **(A)** Kaplan-Meier survival curves of in normal and prediabetic ESCC patients with blood type O. **(B)** Kaplan-Meier survival curves of in normal and prediabetic ESCC patients with blood type A. **(C)** Kaplan-Meier survival curves of in normal and prediabetic ESCC patients with blood type B. **(D)** Kaplan-Meier survival curves of in normal and prediabetic ESCC patients with blood type AB.

Further, the survival status between normal and prediabetic patients with ESCC across the ABO blood groups is illustrated in Figure 2. The cumulative survival rates were significantly higher in normal patients than in prediabetic patients for the blood types O and A (Log-rank test *P* < 0.05) (Figures 2A,B). By contrast, no significance was detected for the blood types B and AB (Figures 2C,D). In view of this divergent observation and to increase statistical power, ESCC patients with the blood types O and A (the blood type B^−^ group) were grouped together, and patients with the blood types B and AB (the blood type B^+^ group) were grouped together in the following analysis.

The results of unadjusted and adjusted Cox proportional hazard regression models for normal and prediabetic patients with ESCC are shown in the Supplemental Table S2 and Table 2, respectively. After adjusting for age, gender, smoking, drinking, body mass index, family cancer history, hypertension, dyslipidemia and TNM stage, risk estimates for ESCC-specific mortality for prediabetic patients relative to normal patients were statistically significant in the blood type B^−^ group (HR: 1.71; 95% CI: 1.33–2.20; *P* < 0.001), but not in the blood type B^+^ group (HR: 1.12; 95% CI: 0.77–1.64; *P* = 0.5544; Table 2). Besides, aging (HR: 1.01; 95% CI: 1.00–1.02; *P* = 0.0387) and dyslipidemia (HR: 1.14; 95% CI: 1.14–1.68; *P* = 0.0010) were also associated with the significant risk of ESCC-specific mortality in the blood type B^−^ group, but not in the blood type B^+^ group (Table 2). By contrast, poor histological differentiation was statistically significant relative to well histological differentiation in the blood type B^+^ group (HR: 1.85; 95% CI: 1.18–2.92; *P* = 0.0079), but not in the blood type B^−^ group (HR: 1. 35; 95% CI: 0.97–1.87; *P* = 0. 0740; Table 2). Although smoking, drinking and family cancer history in prediabetic patients were significantly associated with an increasing risk of ESCC-specific mortality in univariate analyses (Supplemental Table 2), no significance was attained after adjusting for the rest confounders (Table 2).

**Table 2:** Adjusted COX models for normal fasting glucose and prediabetic patients with blood type B+ and B–.

**Risk factors**		**Blood type B–**		**Blood type B**+		***P*-values between two HRs**
		**HR (95% CI)**	***P*-value**^†^	**HR (95% CI)**	***P*-value**^†^	
Prediabetes	Normal	Ref		Ref		
	Prediabetes	1.71 (1.33–2.20)	<0.0001	1.12 (0.77–1.64)	0.5544	0.0713
Age		1.01 (1.00–1.02)	0.0387	1.00 (0.98–1.01)	0.9024	0.1985
Sex	Male	Ref		Ref		
	Female	0.76 (0.57–1.00)	0.0482	0.65 (0.44–0.97)	0.0364	0.5537
Smoking		1.05 (0.83–1.33)	0.7035	0.94 (0.67–1.32)	0.7328	0.6186
Drinking		1.09 (0.85–1.41)	0.4840	0.81 (0.55–1.20)	0.3005	0.2116
Family cancer history		0.94 (0.71–1.24)	0.6682	1.03 (0.70–1.51)	0.8960	0.7213
Body mass index		0.98 (0.95–1.01)	0.2048	1.02 (0.97–1.07)	0.3870	0.1493
Tumor-node-metastasis stage	I/II	Ref		Ref		
	III/IV	3.41 (2.71–4.29)	<0.0001	3.47 (2.49–4.84)	<0.0001	0.9239
Invasion depth	T1/T2	Ref		Ref		
	T3/T4	2.16 (1.69–2.75)	<0.0001	2.05 (1.43–2.93)	<0.0001	0.8137
Regional lymph node metastasis	N0	Ref		Ref		
	N1	2.35 (1.82–3.03)	<0.0001	2.89 (1.99–4.22)	<0.0001	0.3653
	N2/N3	3.88 (3.03-4.97)	<0.0001	4.52 (3.15-6.50)	< .0001	0.4921
Distant metastasis	Negative	Ref		Ref		
	Positive	2.98 (2.41–3.68)	<0.0001	2.96 (2.19–4.01)	<0.0001	0.9793
Histological differentiation	Well	Ref		Ref		
	Moderate	1.21 (0.91–1.60)	0.1851	1.39 (0.92–2.08)	0.1167	0.5921
	Poor	1.35 (0.97–1.87)	0.0740	1.85 (1.18–2.92)	0.0079	0.2671
Embolus	Negative	Ref		Ref		
	Positive	2.12 (1.71–2.64)	<0.0001	1.94 (1.37–2.76)	0.0002	0.6742
Tumor size		1.15 (1.10–1.20)	<0.0001	1.19 (1.12–1.26)	<0.0001	0.4398
Number of regional lymph node metastasis		1.07 (1.05–1.08)	<0.0001	1.12 (1.09–1.15)	<0.0001	0.0026
Hypertension		1.13 (0.90–1.43)	0.2806	1.15 (0.83–1.59)	0.4134	0.9585
Dyslipidemia		1.39 (1.14–1.68)	0.0010	0.93 (0.71–1.22)	0.6142	0.0202

*HR, hazard ratio; 95% CI, 95% confidence interval*.

† *P-values were calculated after adjusting for age, sex, smoking, drinking, body mass index, family cancer history, hypertension, dyslipidemia, and TNM stage, and the risk prediction of each adjusted factor was calculated by adjusting for the other factors*.

### Prognostic nomogram

**Figure 3:**
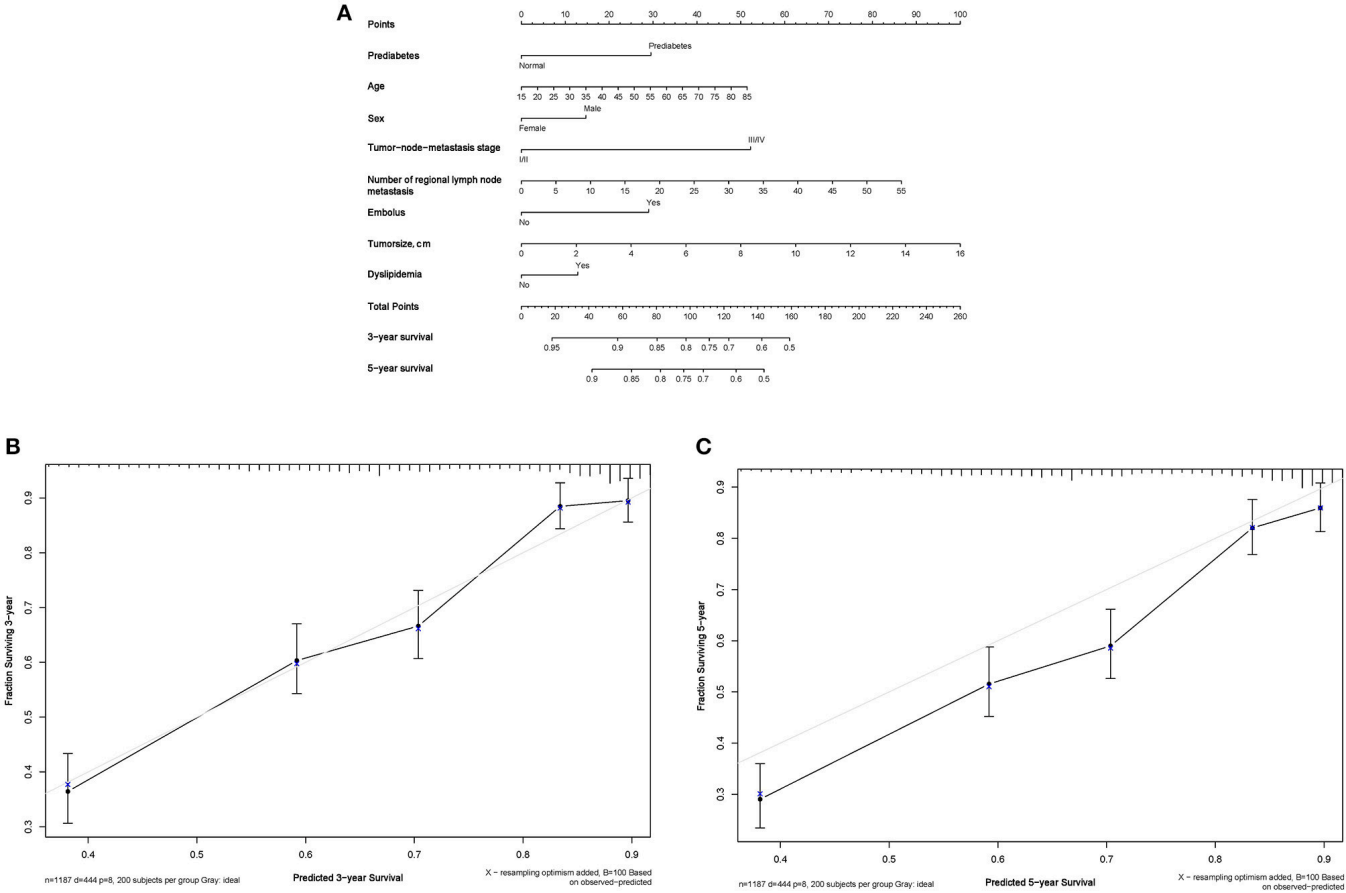
Nomogram plot and associated calibration curve in ESCC patients with the “B-” blood type. **(A)** Nomogram plot of 3-year and 5-year survival in ESCC patients with the “B-” blood type. **(B)** Calibration curve of 3-year survival in ESCC patients with the “B-” blood type. **(C)** Calibration curve of 5-year survival in ESCC patients with the “B-” blood type.

Shown in Figure 3A is the prognostic nomogram predicting the probability of 3- and 5-year survival after three-field lymphadenectomy in the blood type B^−^ group based on the significant results in Table 2, with calibration curves portrayed in Figures 3B,C. Three-year survival calibration curve was perfectly fitted, indicating that the presurgical risk factors selected can better predict the risk of the ESCC-specific mortality (C-index: 0.714).

## Discussion

In this present study, we aimed to investigate whether prediabetes was interacted with the ABO blood group in the prediction of ESCC-specific mortality among 1,857 normal and prediabetic patients over a 15-year follow-up period. The key finding is that prediabetes can predict the significant risk of ESCC-specific mortality in Chinese Han patients with the blood types O and A, while the prediction was nonsignificant for the blood types B and AB, implying an interaction between prediabetes and the blood B^−^ group. To the best of our knowledge, this is thus far the first study that has evaluated the prognosis of prediabetes and the ABO blood type for postsurgical ESCC-specific morality. Extending the previous findings on the association of blood type with diabetes mellitus(27) and cancer (28–30), this study highlights the close monitoring of blood glucose at the prediabetes stage in postsurgical ESCC patients, especially with the blood types O and A.

According to a large national survey, the prevalence of prediabetes was 35.7% in China, meaning that over 460 million Chinese adults have prediabetes (31). It is widely accepted that prediabetes is a significant risk factor for a wide range of clinical endpoints, including cardiovascular diseases, cognitive dysfunction, microvascular diseases, blood pressure abnormalities, metabolic syndrome and cancer (32, 33). Prediabetes is recognized as a silent killer, and it places a major burden on individual and public health. Given these issues, the clinical implication of this present study is to make a large proportion of ESCC patients realize the importance of prediabetes as an early warning sign of a poor prognosis after radical resection in the future, and to attach top priority to keep glucose within physiologically acceptable levels to prolong the survival of postsurgical ESCC patients.

It is also of interest to note that the prediction of prediabetes for ESCC-specific morality was hinged on different ABO blood types, as revealed by the interaction analysis of this study. The ABO blood group system was first coined by Karl Landsteiner, including A, B, O, and AB blood types (34). Accruing evidence indicates the involvement of the ABO blood group system in the development or prognosis of many malignancies, including esophageal cancer. For instance, a study by Qin and colleagues showed that ESCC patients with the blood type AB had a significantly worse overall survival than patients with the other blood types, especially in patients with negative lymph nodes metastasis (29). Additionally, Sun and colleagues found that the blood types B and O can significantly predict the mortality of ESCC patients who ever smokers (28). Also, prediabetes and metabolic syndrome were identified as risk factors for cancer development and mortality (4, 33). However, no evidence is currently available on the interaction between prediabetes and the ABO blood type in the literature. Interestingly, we for the first time observed that prediabetes was significantly interacted with the absence of the blood type B carriers, which can aid clinical prognostication and facilitate individualized evaluation of ESCC patients with prediabetes and the blood types O and A.

Finally, some possible limitations should be acknowledged for this study. First, only fasting blood glucose was available for us, and it will be informative for the performance of oral glucose tolerance test when assessing prediabetes. Second, data on drug regimens such as statins were also not available, which precluded us to explore their contributory or confounding roles in ESCC-specific mortality. Third, all study patients were consecutively enrolled within 10 years, and during this period technical advances might beget selection bias. Fourth, all patients are Han Chinese, which may confine the generalizability of our results to other nationalities or races.

Taken together, our findings indicate that prediabetes can predict the significant risk of ESCC-specific mortality in Chinese Han patients with the blood types O and A, while the prediction was nonsignificant for the blood types B and AB. For practical reasons, our findings call for the screening of prediabetes in ESCC patients with the O or A blood type and improve management of blood glucose, which may help improve the survival outcomes. More prospective studies are warranted to certify our findings.

## Author contributions

DH, FP, XZ, JJ, and WN planned and designed the study, and directed its implementation. DH, FP, and XZ drafted the protocol. GC, BL, HZ, and YX obtained statutory and ethics approvals. DH, XL, GC, BL, HZ, and YX contributed to data acquisition. WN, FP, GF, and XZ conducted statistical analyses. WN, DH, FP, XZ had access to all raw data. DH, FP, and WN did the data preparation and quality control. GF, FP, JJ, and WN wrote the manuscript. All authors read and approved the final manuscript prior to submission.

### Conflict of interest statement

The authors declare that the research was conducted in the absence of any commercial or financial relationships that could be construed as a potential conflict of interest.
