# Supplementary material for: Interaction Between Prediabetes and the ABO Blood Types in Predicting Postsurgical Esophageal Squamous Cell Carcinoma-Specific Mortality: The FIESTA Study
Source: Front Oncol. 2018 Oct 23;8:461. doi: 10.3389/fonc.2018.00461 (PMC6206301; doi:10.3389/fonc.2018.00461)
Supplement: Supplementary file 1 [file Data_Sheet_1.PDF]

Supplemental Table S1. The Interactive analyses of COX models between prediabetes and blood types

Interactive factors	Unadjusted Model			Adjusted Model		
	Parameter Estimate	Standard Error	P value	Parameter Estimate	Standard Error	P value†
Prediabetes						
Normal	Ref		-	Ref		-
Prediabetes	0.7007	0.1656	<.0001	0.6316	0.1695	0.0002
Blood types						
O	Ref		-	Ref		-
A	0.1268	0.1020	0.2137	0.0811	0.1066	0.4466
B	0.1496	0.1060	0.1583	0.1272	0.10940	0.2449
AB	0.2045	0.1741	0.2402	0.1224	0.1816	0.5002
Prediabetes * Blood type O	Ref		-	Ref		-
Prediabetes * Blood type A	-0.1659	0.2468	0.5014	-0.1233	0.2522	0.6248
Prediabetes * Blood type B	-0.5304	0.2574	0.0393	-0.4503	0.2629	0.0867
Prediabetes * Blood type AB	-0.4686	0.5031	0.3516	-0.5522	0.5069	0.2760

† P values were calculated after adjusted for sex, smoking, drinking, body mass index, family cancer history, hypertension, dyslipidemia, and TNM stage.

Supplemental Table S2. Unadjusted COX models for normal fasting glucose and prediabetic patients with blood type B+ and B-

Risk factors		Blood type B-		Blood type B+		P values between two HRs
		HR (95% CI)	P value	HR (95% CI)	P value	
Prediabetes	Normal	Ref		Ref		
	Prediabetes	1.86 (1.46-2.36)	<.0001	1.20 (0.84-1.72)	0.3064	0.0492
Age		1.01 (1.00-1.02)	0.1799	1.00 (0.99-1.02)	0.5849	0.7334
Sex	Male	Ref		Ref		
	Female	0.62 (0.49-0.78)	<.0001	0.53 (0.37-0.77)	0.0006	0.5071
Smoking		1.42 (1.18-1.71)	0.0002	1.18 (0.90-1.54)	0.2235	0.2526
Drinking		1.33 (1.08-1.65)	0.0086	1.06 (0.77-1.46)	0.7152	0.2445
Family cancer history		1.06 (0.81-1.39)	0.6852	0.94 (0.65-1.36)	0.7356	0.6091
Body mass index		0.97 (0.94-1.01)	0.0996	0.99 (0.95-1.04)	0.7141	0.5123
Tumor-node-metastasis stage	I/II	Ref		Ref		
	III/IV	3.88 (3.11-4.85)	<.0001	3.89 (2.81-5.38)	<.0001	0.9916
Invasion depth	T1/T2	Ref		Ref		
	T3/T4	2.43 (1.92-3.08)	<.0001	2.16 (1.53-3.05)	<.0001	0.5753

Risk factors		Blood type B-		Blood type B+		P values between two HRs
		HR (95% CI)	P value	HR (95% CI)	P value	
Regional lymph node metastasis	N0	Ref		Ref		
	N1	2.57 (2.01-3.29)	<.0001	3.18 (2.21-4.58)	<.0001	0.3428
	N2/N3	4.37 (3.45-5.55)	<.0001	5.29 (3.72-7.54)	<.0001	0.3798
Distant metastasis	Negative	Ref		Ref		
	Positive	3.23 (2.63-3.95)	<.0001	3.24 (2.41-4.36)	<.0001	0.9804
Histological differentiation	Well	Ref		Ref		
	Moderate	1.22 (0.93-1.60)	0.1552	1.32 (0.90-1.94)	0.1597	0.7403
	Poor	1.40 (1.02-1.93)	0.0389	1.71 (1.11-2.66)	0.0160	0.4650
Embolus	Negative	Ref		Ref		
	Positive	2.26 (1.83-2.80)	<.0001	2.02 (1.46-2.80)	<.0001	0.5663
Tumor size		1.18 (1.13-1.23)	<.0001	1.20 (1.13-1.27)	<.0001	0.5976
Number of regional lymph node metastasis		1.07 (1.06-1.08)	<.0001	1.12 (1.10-1.15)	<.0001	0.0003
Hypertension		1.11 (0.90-1.37)	0.3423	1.20 (0.88-1.62)	0.2466	0.6878
Dyslipidemia		1.46 (1.21-1.76)	<.0001	1.03 (0.79-1.34)	0.8053	0.0363

Abbreviations: HR, hazard ratio; 95% CI, 95% confidence interval.
